# GB Virus C (GBV-C) Infection in Hepatitis C Virus (HCV) Seropositive Women with or at Risk for HIV Infection

**DOI:** 10.1371/journal.pone.0114467

**Published:** 2014-12-10

**Authors:** Jason T. Blackard, Gang Ma, Jeffrey A. Welge, Caroline C. King, Lynn E. Taylor, Kenneth H. Mayer, Robert S. Klein, David D. Celentano, Jack D. Sobel, Denise J. Jamieson, Lytt Gardner

**Affiliations:** 1 Division of Digestive Diseases, University of Cincinnati College of Medicine, Cincinnati, OH, United States of America; 2 Departments of Psychiatry and Environmental Health, University of Cincinnati College of Medicine, Cincinnati, OH, United States of America; 3 Division of Reproductive Health, Centers for Disease Control and Prevention, Atlanta, GA, United States of America; 4 Miriam Hospital and Department of Medicine, Brown University, Providence, RI, United States of America; 5 Beth Israel Deaconess Medical Center and the Fenway Institute, Boston, MA, United States of America; 6 Division of Infectious Diseases, Mt. Sinai St. Luke’s and Mt. Sinai Roosevelt Hospitals, Mount Sinai School of Medicine, New York, NY, United States of America; 7 Department of Epidemiology, Johns Hopkins Bloomberg School of Public Health, Baltimore, MD, United States of America; 8 Division of Infectious Diseases, School of Medicine, Wayne State University, Detroit, MI, United States of America; 9 Division of HIV/AIDS Prevention, Centers for Disease Control and Prevention, Atlanta, GA, United States of America; UCL Institute of Child Health, University College London, United Kingdom

## Abstract

**Background:**

GB virus C (GBV-C) may have a beneficial impact on HIV disease progression; however, the epidemiologic characteristics of this virus are not well characterized. Behavioral factors and gender may lead to differential rates of GBV-C infection; yet, studies have rarely addressed GBV-C infections in women or racial/ethnic minorities. Therefore, we evaluated GBV-C RNA prevalence and genotype distribution in a large prospective study of high-risk women in the US.

**Results:**

438 hepatitis C virus (HCV) seropositive women, including 306 HIV-infected and 132 HIV-uninfected women, from the HIV Epidemiologic Research Study were evaluated for GBV-C RNA. 347 (79.2%) women were GBV-C RNA negative, while 91 (20.8%) were GBV-C RNA positive. GBV-C positive women were younger than GBV-C negative women. Among 306 HIV-infected women, 70 (22.9%) women were HIV/GBV-C co-infected. Among HIV-infected women, the only significant difference between GBV-negative and GBV-positive women was age (mean 38.4 vs. 35.1 years; p<0.001). Median baseline CD4 cell counts and plasma HIV RNA levels were similar. The GBV-C genotypes were 1 (n = 31; 44.3%), 2 (n = 36; 51.4%), and 3 (n = 3; 4.3%). The distribution of GBV-C genotypes in co-infected women differed significantly by race/ethnicity. However, median CD4 cell counts and log_10_ HIV RNA levels did not differ by GBV-C genotype. GBV-C incidence was 2.7% over a median follow-up of 2.9 (IQR: 1.5, 4.9) years, while GBV-C clearance was 35.7% over a median follow-up of 2.44 (1.4, 3.5) years. 4 women switched genotypes.

**Conclusions:**

Age, injection drug use, a history of sex for money or drugs, and number of recent male sex partners were associated with GBV-C infection among all women in this analysis. However, CD4 cell count and HIV viral load of HIV/HCV/GBV-C co-infected women were not different although race was associated with GBV-C genotype.

## Introduction

GB virus type C (GBV-C), first isolated in 1995, is a single-stranded RNA virus that belongs to the *Flaviviridae* family and is the closest known relative of the hepatitis C virus (HCV) [1]. GBV-C is transmitted efficiently via percutaneous and sexual routes. While male-to-male sex is a highly effective mode of GBV-C transmission [2], there is ample evidence that GBV-C can be transmitted heterosexually and perinatally as well [3]. The reported prevalence of GBV-C RNA ranges from 14 to 45% in HIV-infected persons [4]. However, the prevalence of GBV-C RNA is highly variable across distinct at-risk groups [4]. In a cross-sectional analysis of HIV-infected patients attending an urban HIV clinic, GBV-C RNA was most common among men who have sex with men (MSM) (35%) but less common among injection drug users (IDUs) (22%) or heterosexual men/women (22%) [5]. Furthermore, persistent GBV-C viremia is highest in MSM and is associated with an increased number of partners [2]. However, no trend between GBV-C persistence and number of partners was found among female sex workers. These data imply that gender and/or behavioral differences may lead to different rates of GBV-C infection and/or viral persistence. Several international studies have examined perinatal transmission of GBV-C or the impact of GBV-C infection on HIV transmission or disease progression in pregnant women [6]–[13]. For instance, in a large study of pregnant women, GBV-C infection was associated with increasing number of lifetime sexual partners, IDU, and HIV infection, while GBV-C clearance was associated with increasing age, more than 10 lifetime sexual partners, and no HIV infection [14]. Sexual transmission of GBV-C has been examined as well [2], [15], [16]. We reported previously that active GBV-C infection was more common in men than women and that more women than men had no evidence of GBV-C infection [17]. In health clinic attendees in the US, a higher prevalence of GBV-C occurred in subjects currently seeking STD treatment, while non-white race was associated with GBV RNA positivity [18]. GBV-C infection has been investigated in HIV-positive pregnant women in the US but did not include evaluation of factors associated with GBV-C infection [19].

Several groups have reported beneficial effects of GBV-C viremia on HIV disease [20]–[27]. At the population level, at least 7 GBV-C genotypes exist based upon phylogenetic analysis of the 5′ untranslated region (UTR) [28], [29]. The existence of multiple GBV-C genotypes has led several authors to suggest that differences in GBV-C strains circulating within populations might affect their impact on HIV disease [4], [30], [31]. For example, Muerhoff *et al.* reported that CD4 cell counts were lower in HIV co-infected patients infected with GBV-C genotype 2a than patients with genotype 2b [31]. We found that among a cohort with HIV/HCV/GBV-C triple infection, GBV-C genotype 2 was associated with higher CD4 cell counts compared to GBV-C genotype 1 [17]. While similar findings have also been reported in a Brazilian cohort [32], a comparison of genotype 2 versus non-2 infections in an Australian cohort observed no such difference in CD4 cell counts [33]. GBV-C viral load may also differ by genotype [34]. Thus, it is possible that GBV-C genotype could at least partially account for the beneficial association between GBV-C replication and HIV disease progression but requires further investigation in larger cohorts with multiple circulating GBV-C genotypes.

Investigations of GBV-C in women are generally limited to cross-sectional prevalence studies and perinatal transmission studies. Large longitudinal studies specifically addressing the prevalence and possible effects of GBV-C co-infection and GBV-C genotypes on the natural history of HIV infection in women are rare. Thus, there is limited information regarding the sociodemographic factors associated with GBV-C infection in women or racial minorities, particularly in the US. Therefore, the current study evaluated the rate of GBV-C RNA detection, GBV-C genotype distribution, and the GBV-C incidence and clearance rates in a large cohort study of HIV-infected and high-risk, HIV-uninfected women in the US with a substantial proportion of racial minorities. In understudied and/or disadvantaged populations such as women, ethnic minorities, and IDUs in which HIV-infected individuals may not have access to antiretroviral therapy, a better understanding of the epidemiology of GBV-C infection, as well as the anti-HIV effects of GBV-C infection, may ultimately result in novel therapeutic strategies.

## Materials and Methods

### Study population and sample selection

From 1993 until 2000, a prospective natural history study of HIV infection - the HIV Epidemiologic Research (HER) Study - was conducted in US women [35]. 871 HIV-infected women and 438 demographically similar, uninfected women were recruited from 4 sites: Baltimore, Maryland; Bronx, New York; Providence, Rhode Island; and Detroit, Michigan. By study design, one-half reported injection drug use (IDU), while the other half reported only sexual risk behavior for HIV. Women were assessed at 6-month intervals for up to 14 study visits. Women with a clinical AIDS diagnosis or any AIDS-defining opportunistic infections were ineligible for enrollment. Among the 871 HIV-infected women enrolled in the HERS cohort, 233 had a CD4 cell count of ≤200 cells/mm^3^ at baseline. At study entry, ∼30% of HIV-infected women received monotherapy or dual therapy, and none received HAART [36]. As described elsewhere, hepatitis C virus (HCV) serostatus was determined using the Abbott HCV EIA 2.0 or Ortho HCV version 3.0 ELISA [37]. In the original cohort, the seroprevalence of HCV was 56.5%, with rates of 48.0% and 60.8% among HIV-uninfected and HIV-infected women, respectively. Less than 1% received any HCV therapy; therefore, HCV treatment is not likely to confound analyses. Institutional review boards at each participating medical center and at the Centers for Disease Control and Prevention approved the HER study protocol. All participants provided voluntary informed consent prior to sample and clinical data collection.

To facilitate future analyses of the effects of GBV-C infection on liver disease during HIV/HCV co-infection, this analysis was restricted to 438 randomly selected women who were HCV seropositive and had available serum for analysis. The current study population did not differ at baseline from the remaining women in the HERS cohort with respect to race, HIV serostatus, or mean HIV RNA levels. However, women in the current analysis were older than the remaining women (37.0 years at baseline versus 34.0 years [p<0.001] for HIV-positive women and 36.8 years versus 33.1 years [p<0.001] for HIV-negative women). CD4 cell counts at baseline were also significantly different (411.8 cells/mm^3^ for HIV-positive women in the current study versus 365.4 cells/mm^3^ for the remaining HIV-positive HERS women, p = 0.021).

### Detection of GBV-C RNA

Viral RNA was extracted from 140 µl of serum with the QIAmp Viral RNA Mini Kit (QIAGEN, Valencia, CA) following the manufacturer’s instructions. GBV-C RNA was detected by nested RT-PCR using primers corresponding to the 5′ untranslated region (UTR) as described previously [17]. PCR products were analyzed by agarose gel electrophoresis for the presence of a 256-nucleotide band corresponding to nucleotides 107–362 of GenBank accession number AY196904.

### Phylogenetic analysis

GBV-C genotype was determined by population-based amplification of the 5′UTR region as described previously [17]. Sequences were aligned with a database reference using Clustal X 2.1 [38]. The reference sequences used to confirm GBV-C genotype included the following GenBank accession numbers: 1A: U59540 and U59543; 1B: U59549 and U59555; 2A: U59520 and U59521; 2B: U59529 and U59533; 3: U59538 and U59539; 4: AB018667 and AB021287; 5: AY949771 and AF092894; 6: AB003292 and AF177619. The statistical robustness and reliability of the branching order within the phylogenetic tree was confirmed by bootstrap analysis using 1,000 replicates. Additional phylogenetic inference was performed using a Bayesian Markov chain Monte Carlo (MCMC) approach as implemented in the Bayesian Evolutionary Analysis by Sampling Trees (BEAST) v1.7.5 program [39] under an uncorrelated log-normal relaxed molecular clock and the generalized time reversible (GTR) model with nucleotide site heterogeneity estimated using a gamma distribution. The BEAST MCMC analysis was run for a chain length of 200,000,000. All effective sample size values were >100 indicating sufficient sampling. The maximum clade credibility tree was selected from the posterior tree distribution after a 10% burn-in using TreeAnnotator v1.7.5.

### Statistical analysis

Categorical variables were evaluated for an association with GBV-C infection using the chi-squared test or Fisher’s exact test if any expected cell count was less than 5. The means of normally-distributed continuous variables were compared across GBV-C infection status using a t-test and across the three GBV-C genotypes using a one-way analysis of variance. The medians of non-normally distributed continuous variables were compared across GBV-C infection status using the Wilcoxon rank sum test and across genotypes using the Kruskal-Wallis test. All p-values are two-sided. P-values <0.10 are provided, although only p-values <0.05 are considered statistically significant.

## Results

438 women, including 306 HIV-infected and 132 HIV-uninfected women, from the HERS cohort were evaluated for GBV-C RNA. 347 women were GBV-C RNA negative (79.2%), while 91 were GBV-C RNA positive (20.8%) as shown in Table 1. In univariate analysis, GBV-C positive women were younger (p<0.001), less likely to report a history of IDU (p = 0.036), and more likely to have sex partners in the past 6 months (p = 0.037) than GBV-C negative women. GBV-C positive women were also more likely to be HIV-positive than GBV-C negative women, although this difference did not reach statistical significance (p = 0.099).

**Table 1:** Demographic and clinical data for 438 women from the HERS cohort included in the current study.

Characteristic	GBV-C RNAnegative(N = 347)	GBV-C RNApositive(N = 91)	P value
Mean age in years (SD)	38.3 (6.0)	34.7 (6.3)	<0.001
Race (%)			NS
Black	220 (63.4%)	52 (57.1%)	
White	75 (21.6%)	22 (24.2%)	
Hispanic	48 (13.8%)	16 (17.6%)	
Native American/Asian/Other	4 (1.2%)	1 (1.1%)	
Non-white	272 (78.4%)	69 (75.8%)	
White	75 (21.6%)	22 (24.2%)	
Sexual and drug use behaviors			
IDU since 1985 (%)	327 (94.2%)	80 (87.9%)	0.036
IDU in previous 6 months (%)	128 (36.9%)	35 (38.5%)	NS
≥5 sex partners in past 6 months (%)	109 (31.4%)	23 (25.3%)	NS
Ever had sex with male IDU (%) [435]	301/344 (87.5%)	80/91 (87.9%)	NS
Ever had sex with partnerknown/suspected HIV+ (%) [435]	179/344 (52.0%)	48/91 (52.8%)	NS
Ever had sex for money or drugs (%)	200 (57.6%)	42(46.2%)	0.050
Male sex partners in previous 6 months (%)[436]			
0	107/345 (31.0%)	19 (20.9%)	
1–10	229/345 (66.4%)	72 (79.1%)	
>10	9/345 (2.6%)	0	0.037
Currently using hormonal contraceptives (%)	11 (3.2%)	5 (5.5%)	NS
Currently using condoms (%)[309 sexually active]	170/238 (71.4%)	50/71 (70.4%)	NS
Ever been pregnant (%)	326 (94.0%)	87 (95.6%)	NS
Currently pregnant (%) [435]	3/344 (0.9%)	0	NS
Currently using alcohol (%)	200 (57.6%)	52 (57.1%)	NS
All-cause mortality (%)			NS
1993–1996	24 (6.9%)	8 (8.8%)	
1997–2000	39 (11.2%)	11 (12.1%)	
HIV positive (%)	236 (68.0%)	70 (76.9%)	0.099
HBsAg positive (%) [345]	13/278 (4.7%)	3/67 (4.5%)	NS

In parentheses are percentages unless otherwise noted. Numbers in brackets denote women with available data. P-values <0.10 are provided, although only p-values <0.05 are considered statistically significant. NS = not significant (p≥0.10); SD = standard deviation.

306 women with HIV were evaluated for GBV-C RNA, and 70 (22.9%) were HIV/GBV-C co-infected. As shown in Table 2, the only significant difference between HIV-infected/GBV-negative and HIV-infected/GBV-positive women was age (p<0.001) with dually infected women being younger. Median baseline CD4 cell count and plasma HIV RNA, as well as the proportion of women on antiretroviral therapy, were similar between HIV-positive women with and without GBV-C RNA.

**Table 2:** Demographic and clinical data for 306 HIV-positive women from the HERS cohort included in the current study.

Characteristic	GBV-C RNAnegative(N = 236)	GBV-C RNApositive(N = 70)	P value
Mean age in years (SD)	38.4 (5.8)	35.1 (6.2)	<0.001
Race (%)			NS
Black	156 (66.4%)	43 (61.4%)	
White	43 (18.3%)	14 (20.0%)	
Hispanic	34 (14.5%)	12 (17.1%)	
Native American/Asian/Other	2 (0.8%)	1 (1.4%)	
Non-white	192 (81.7%)	56 (80.0%)	
White	43 (18.3%)	14 (20.0%)	
Sexual and drug use behaviors			NS
IDU since 1985 (%)	219 (93.2%)	61 (87.1%)	
IDU in previous 6 months (%)	77 (32.6%)	24 (34.3%)	
≥5 sex partners in past 6 months (%)	64 (27.2%)	15 (21.4%)	
Ever had sex with male IDU (%) [303]	208/233 (89.3%)	63/70 (90.0%)	
Ever had sex with partner known/suspected HIV+ (%) [304]	134/234 (57.3%)	42/70 (60.0%)	
Ever had sex for money or drugs (%)	132 (56.2%)	32 (45.7%)	
Male sex partners in previous 6 months (%) [304]			NS
0	83 (35.2%)	17 (24.3)%	
1–10	149 (63.1%)	53 (75.7)%	
>10	4 (1.7%)	0	
Currently using hormonal contraceptives (%)	7 (3.0%)	4 (5.7%)	NS
Currently using condoms (%) [206]	119/153 (77.8%)	39/53 (73.6%)	NS
Ever been pregnant (%)	221 (94.0%)	67 (95.7%)	NS
Currently pregnant (%) [304]	2/234 (0.9%)	0/70 (0%)	NS
Currently using alcohol (%)	129 (54.7%)	38 (54.3%)	NS
All-cause mortality (%)			NS
1993–1996	23 (9.8%)	8 (11.4%)	
1997–2000	37 (15.7%)	11 (15.7%)	
CD4 cell count (%) [296]			NS
<200	39/229 (17.0%)	9/67 (13.4%)	
200–499	117/229 (51.1%)	40/67 (59.7%)	
≥500	73/229 (31.9%)	18/67 (26.9%)	
Median CD4 cell count in cells/uL (IQR) [296]	382 (251, 571)	413 (267, 536)	NS
Median log_10_ HIV RNA in copies/ml(IQR) - all women with data [301]	3.24 (2.65, 3.90)	3.46 (2.81, 3.85)	NS
Median log_10_ HIV RNA in copies/ml(IQR) - only women not on ART [202]	3.24 (2.65, 4.06)	3.47 (2.81, 3.83)	NS
Currently using ART (%)	74 (31.4%)	27 (38.6%)	NS
HBsAg positive (%) [246]	7/192 (3.7%)	2/54 (3.7%)	NS

In parentheses are percentages unless otherwise noted. Numbers in brackets denote women with available data. P-values <0.10 are provided, although only p-values <0.05 are considered statistically significant. NS = not significant (p≥0.10); SD = standard deviation; IQR = interquartile range.

**Figure 1:**
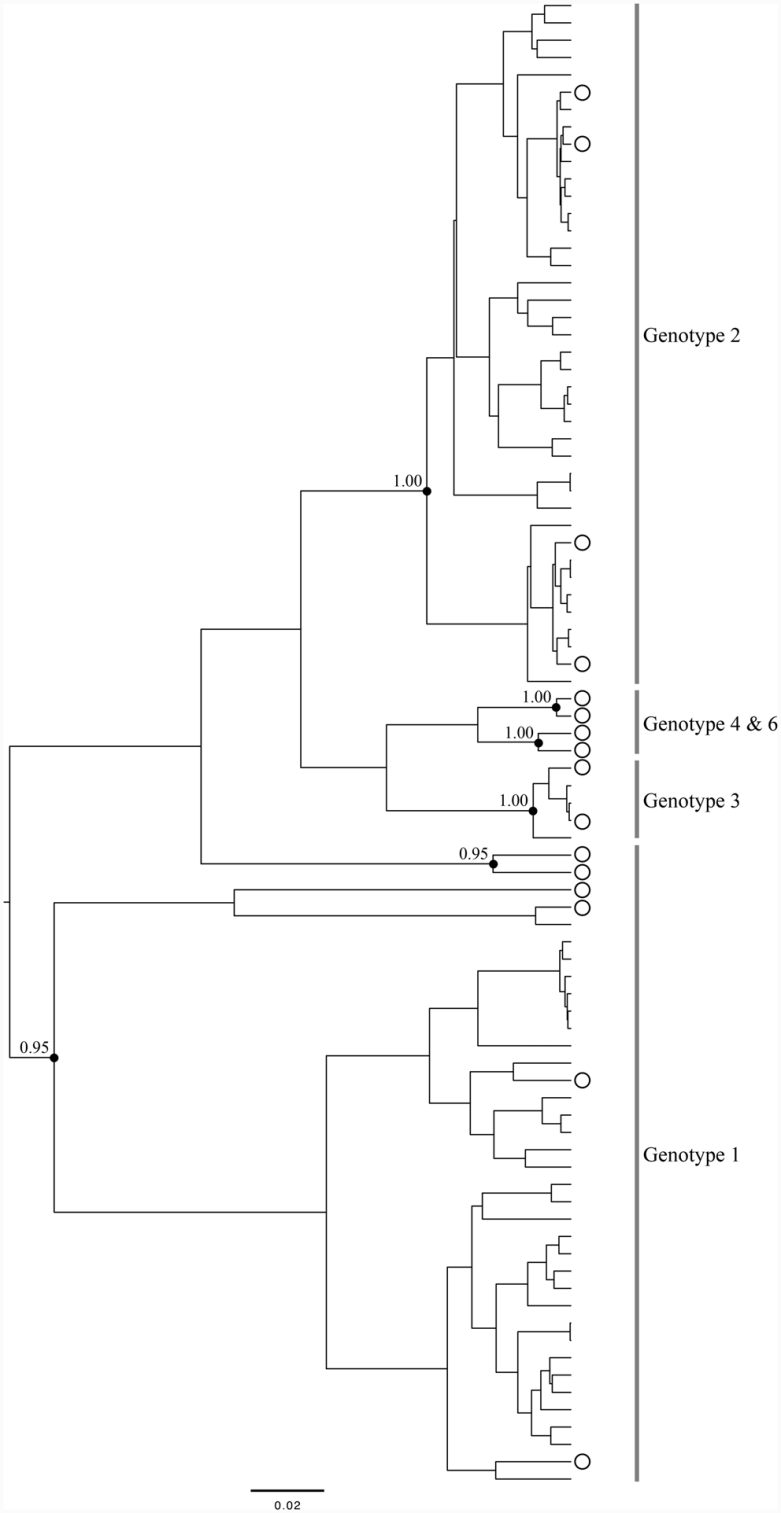
Phylogenetic tree based on consensus 5′UTR sequences for 70 HIV/GBV-C co-infected women. GenBank reference sequences are indicated by open circles. Relevant posterior probabilities (out of 1.00) are shown. The scale bar indicates 0.02 nucleotide substitutions per site.

Previous studies suggest a potential role for GBV-C genotype in modulating HIV disease; therefore, we determined GBV-C genotype among HIV/GBV-C co-infected women (Fig. 1). Based on analysis of the 5′UTR, the GBV-C genotypes were 1 (n = 31; 44.3%), 2 (n = 36; 51.4%), and 3 (n = 3; 4.3%). The distribution of GBV-C genotypes in co-infected women was significantly different among racial groups (p = 0.003) with Hispanics being more likely to be infected with genotype 1 and whites being less likely to be infected with this genotype (Table 3). There was also a suggestion of distinct GBV-C genotype distributions in previous IDUs versus women that were never IDUs, with the latter being predominantly infected with genotype 2 (p = 0.080). However, median baseline CD4 cell counts (interquartile range [IQR]) were not markedly different across the three GBV-C genotypes: 400 (IQR: 277, 468), 408 (IQR: 267, 577), and 490 (IQR: 222, 490) cells/mm^3^ for women infected with genotypes 1, 2, and 3, respectively. Moreover, median baseline log_10_ HIV RNA levels were similar across genotypes: 3.32 (IQR: 2.79, 4.14), 3.48 (IQR: 2.78, 3.83), and 3.46 (IQR: 2.86, 4.56) copies/mL for genotypes 1, 2, and 3, respectively.

**Table 3:** Demographic and clinical data 70 HIV/GBV-C co-infected women from the HERS cohort included in the current study.

Characteristic	GBV-C genotypes			
	Type 1	Type 2	Type 3	P-value
	(n = 31)	(n = 36)	(n = 3)	
Median age in years (SD)	34.8 (5.1)	35.4 (6.5)	35.9 (13.8)	NS
Race (%)				
Black	20 (64.5%)	22 (61.1%)	1 (33.3%)	0.003
White	1 (3.2%)	11 (30.6%)	2 (66.7%)	
Hispanic	9 (29.0%)	3 (8.3%)	0	
Native American/Asian/Other	1 (3.2%)	0	0	
IV drug use				0.080
Never	1 (3.2%)	8 (22.2%)	0	
Previously	30 (96.8%)	28 (77.8%)	3 (100%)	
All-cause mortality				NS
1993–1996	5 (16.1%)	2 (5.6%)	1 (33.3%)	
1997–2000	4 (12.9%)	7 (19.4%)	0	
CD4 cell count [69]				NS
<200	4 (12.9%)	5/35 (14.3%)	0	
200–499	19 (61.3%)	18/35 (51.4%)	3 (100.0%)	
≥500	8 (25.8%)	12/35 (34.3%)	0	
Median CD4 cell count in cells/uL(IQR) [67]	400 (277, 468)	408 (267, 577)	490 (222, 490)	NS
Median log_10_ HIV RNA in copies/mL(IQR) [69]	3.32 (2.79, 4.14)	3.48 (2.78, 3.83)	3.46 (2.86, 4.56)	NS
Median log_10_ HIV RNA in copies/mL(IQR) - only women not on ART [42]	3.34 (2.81, 4.17)	3.48 (2.78, 3.73)	4.01 (3.46, 4.56)	NS
Currently using ART (%)	14 (45.2%)	12 (33.3%)	1 (33.3%)	NS
HBsAg positive (%) [54]	1 (4.2%)	1 (3.7%)	0	NS

In parentheses are percentages unless otherwise noted. Numbers in brackets denote women with available data. P-values <0.10 are provided, although only p-values <0.05 are considered statistically significant. NS = not significant (p≥0.10); SD = standard deviation; IQR = interquartile range.

193 women were tested for GBV-C RNA at 2 time points. Of the 151 women who were GBV-C negative at their first visit and had a subsequent GBV-C result, 4 (2.7%) became GBV-C RNA positive over a median follow-up of 2.9 (IQR: 1.5, 4.9) years. Of the 42 women who were initially GBV-C RNA positive, 15 (35.7%) became GBV-C RNA negative at the second test over a median follow-up of 2.44 (1.4, 3.5) years. Of the 27 women who were positive at both the first and second test points, 4 (14.8%) switched genotypes: 2 from genotype 1 to genotype 2b, 1 from genotype 2b to genotype 1, and 1 from genotype 2a to genotype 1.

## Discussion

Multiple studies have demonstrated a beneficial effect of GBV-C viremia on HIV disease [20], [22]–[25]. For instance, Tillman *et al.* reported that longer AIDS-free survival, higher CD4 cell counts, and lower plasma HIV viral loads were associated with the presence of GBV-C RNA. Moreover, an inverse correlation between the GBV-C viral load and the HIV viral load – but not CD4 cell count – was observed [22]. Xiang *et al.* further demonstrated prolonged survival among HIV-positive persons co-infected with GBV-C [20]. HIV replication was also inhibited by GBV-C co-infection in peripheral blood mononuclear cell cultures. Using data from the Multicenter AIDS Cohort Study, Williams *et al.* examined the effect of GBV-C infection measured 12–18 months after HIV seroconversion in men but did not find a statistically significant protective effect [25]. However, when a similar analysis was performed using data on GBV-C infection measured 5–6 years after HIV seroconversion, a statistically significant protective effect of GBV-C co-infection was observed. Bjorkman *et al.* reported that loss of GBV-C RNA during follow-up was associated with the poorest survival [21], a finding also confirmed in other studies [25], [26]. Nevertheless, prolonged survival in GBV-C/HIV co-infected persons has not been observed in all studies [21], [26], [40]–[42]. Due to the potential importance of persistence of GBV-C viremia, a meta-analysis [43] was conducted to synthesize data from eight prospective studies of HIV-positive persons in which GBV-C RNA was determined and all-cause death was determined. There was no conclusive evidence for an association between survival and GBV-C infection *early* in HIV disease, but when GBV-C infection was present *later* in HIV disease, a significant reduction in mortality was observed. Studies in the highly active antiretroviral therapy (HAART) era have found that a complete virologic response to HAART was more frequent in patients co-infected with GBV-C, independent of baseline CD4 cell count and plasma HIV RNA level [27]. However, 87% of participants were male; therefore, whether GBV-C RNA is an independent predictor of the virologic response to HAART in women remains to be determined. Collectively, these studies suggest a beneficial effect of GBV-C co-infection even among HAART-treated individuals, although the beneficial effects of GBV-C have not been confirmed in all studies [44]. Although the mechanism remain unclear, a number of biological mechanisms could explain the impact of GBV-C on HIV disease, including downregulation of HIV entry receptors, increased chemokine expression, interference with HIV binding and/or fusion, activation of interferon-stimulated genes, cytokine polarization, altered expression of T lymphocyte activation markers, protection from Fas-mediated apoptosis, and (reviewed in [45]). Further is study is required to elucidate the mechanisms of interaction between HIV and GBV-C.

Male-to-male (MSM) sex is an effective mode of GBV-C transmission, although GBV-C is transmitted heterosexually and perinatally as well [2], [3]. Importantly, persistent GBV-C infection is highest in MSM, and increased partner number is associated with increased GBV-C infection [2]; however, the same association is not observed among female sex workers. In a cross-sectional analysis of HIV-infected patients, GBV-C RNA was more common among MSM than heterosexual men/women [5]. To date, investigations of GBV-C in women are generally limited to cross-sectional prevalence studies and/or perinatal transmission studies with the GBV-C prevalence ranging from 11% to 37% [2], [5]–[7], [9], [15], [16], [19].

In the current study, we examined the associations between GBV-C infection and HIV infection in HCV seropositive women with a significant proportion of racial/ethnic minorities. Our findings suggest that GBV-C infection is common among women with or at risk for HIV infection. Age, injection drug use, a history of sex for money or drugs, and number of recent male sex partners were associated with GBV-C infection among all women in this analysis. HIV positivity was associated with increased likelihood of GBV-C infection, although this did not reach statistical significance. These findings agree with that of Bhanich Supapol *et al*. who reported similar risk factors for GBV-C infection in a cohort of Thai women [14]. We also found that GBV-C genotype distribution differed by racial group and possibly by IDU status among HIV/GBV-C co-infected women. Surprisingly, we did not observe a significant association of GBV-C RNA or GBV-C genotype with HIV disease severity as measured by CD4 cell count or HIV viral load. These disparate results may reflect demographic differences between populations or insufficient statistical power to observe minor differences between GBV-C infected versus uninfected individuals. Moreover, while the majority of GBV-C genotyping studies have utilized population-based amplification of the 5′UTR [46], sequencing the 5′UTR cannot efficiently discriminate GBV-C subtypes (i.e., 1a versus 1b) in every instance.

This study has several limitations. First, the sample size may not provide sufficient power to detect small differences between GBV-C RNA positive and negative individuals. Moreover, given the genotype distribution within this population, we were only able to meaningfully compare GBV-C genotype 1 to genotype 2. Second, this analysis included GBV-C RNA testing at only two time points per woman and did not evaluate GBV-C at all time points available per woman. Thus, the number of GBV-C incident and/or cleared infections may be underestimated, and GBV-C testing of additional time points is warranted. Third, testing for anti-E2 antibodies – indicative of past infection – was not performed, although several studies suggest that E2 antibodies are not a reliable marker of previous GBV-C infection in immunocompromised individuals [17], [47]. Fourth, given the initial inclusion/exclusion criteria of the HER Study, it is unclear if our findings are generalizable to all women or to populations outside of the US. Fifth, GBV-C infection was not associated with decreased immunodeficiency in this analysis; however, this may reflect the low frequency of severe immunodeficiency in these women. As noted above, a meta-analysis concluded that there was limited evidence for an association between *early* GBV-C infection and HIV-related survival. In contrast, a significant reduction in mortality was observed when GBV-C infection was present *later* in HIV disease [43]. Additionally, loss of GBV-C RNA has also been associated with accelerated HIV disease [21], [25], [42]. Thus, longitudinal analyses in women with more advanced immunodeficiency are necessary to evaluate the effects of persistent GBV-C co-infection on HIV disease progression in women. Furthermore, epidemiologic characterization of GBV-C infection and its anti-HIV effects may ultimately result in novel therapeutic strategies in understudied and/or disadvantaged populations such as women, ethnic minorities, and IDUs.
